# Use of *Ulva reticulata* as a growth supplement for tomato (*Solanum lycopersicum*)

**DOI:** 10.1371/journal.pone.0270604

**Published:** 2022-06-27

**Authors:** Nor Jawahir Abu, Japar Sidik Bujang, Muta Harah Zakaria, Shahrizim Zulkifly

**Affiliations:** 1 Department of Biology, Faculty of Science, Universiti Putra Malaysia, UPM Serdang, Selangor Darul Ehsan, Malaysia; 2 Department of Aquaculture, Faculty of Agriculture, Universiti Putra Malaysia, UPM Serdang, Selangor Darul Ehsan, Malaysia; 3 International Institute of Aquaculture and Aquatic Sciences (I-AQUAS), Universiti Putra Malaysia (UPM), Port Dickson, Negeri Sembilan, Malaysia; Bahauddin Zakariya University, PAKISTAN

## Abstract

Mass proliferation and accumulation of the green macroalga *Ulva reticulata* are problems in coastal areas and affect other ecosystems, such as those involving seagrasses. In the absence of any intervention, the decomposition of these macroalgae over time can disrupt the balance of recipient ecosystems. Attention has been given to the potential use of *U*. *reticulata* as a supplier of nutrients for crop species such as tomatoes as a possible solution to the buildup of this unusable seaweed species, which is usually left to decompose in affected seagrass ecosystems; this is the case in the Merambong seagrass meadow in the Sungai Pulai estuary in Gelang Patah, southwestern Johor, Malaysia. We analyzed the macro- and micronutrient contents in *U*. *reticulata* to determine nutrient availability. We also performed greenhouse studies to test the effects of crude extracts from dried *U*. *reticulata*-Extract “A” and fresh *U*. *reticulata*-Extract “B” on plant growth, total yield, and quality vine-ripened fruits. Compared to other seaweed extracts used as plant growth promoters, *U*. *reticulata* extracts have higher nitrogen (N), manganese (Mn), zinc (Zn), and iron (Fe) contents. The application of 30% Extracts “A” and “B” and 50% Extracts “A” and “B” significantly affected tomato plant height. However, extract concentrations that promoted plant height and hastened flowering and fruiting did not increase total fruit yields. Both treatments that positively affected tomato plant height and hastened flowering and fruiting resulted in increased contents of total soluble solids (TSS), beta-carotene, lycopene, ascorbic acid and total titratable acidity (TTA) in the vine-ripened fruits. Agronomically, the application of 5% Extracts “A” and “B”, 10%-20% Extracts “A” and “B”, and 50% Extract “A” doubled the total yield compared to those of the control, and 40% Extract “A” resulted in the highest total fruit yield. In general, tomato plants responded well to Extract “A” than Extract “B” and presented good total fruit yield and quality.

## Introduction

**Fig 1:**
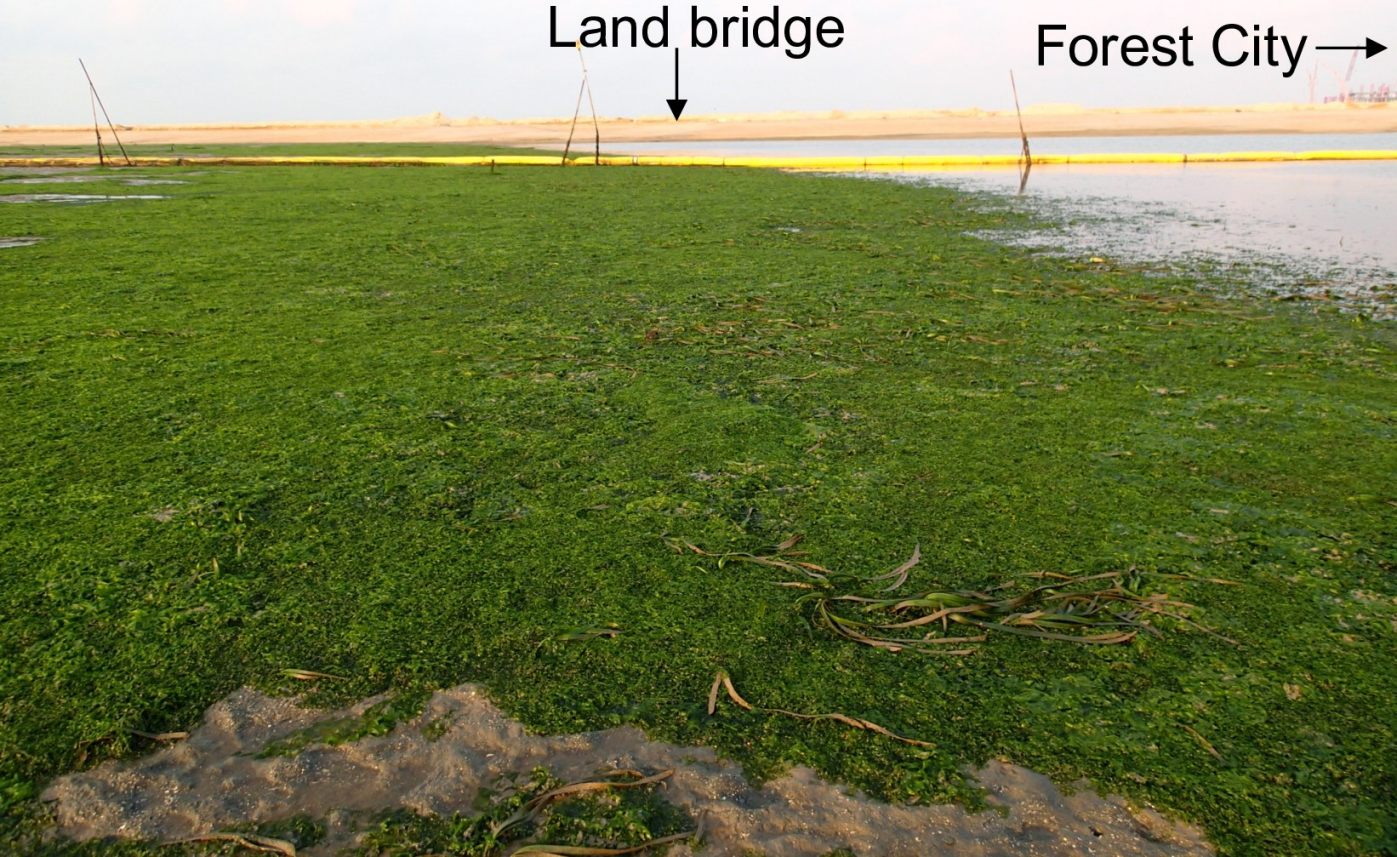
Merambong seagrass meadow near the Forest City and *U*. *reticulata* mass proliferation and accumulation within the seagrass ecosystem.

Seaweed or macroalgal blooms occur worldwide, causing changes in food webs and the loss of ecosystem function and services [1–3]. For example, sand enrichment for beach expansion, which has occurred in Forest City along the Gelang Patah coastline in southwestern Johor, Malaysia, caused mass proliferation and accumulation of *Ulva reticulata* Forsskal. Excessive growth of the *U*. *reticulata* occurred in response to the oversupply of nutrients from the abovementioned activities. The proliferation caused changes in the environment and seagrass ecosystem structure (Fig 1). Attention has given to the potential use of *U*. *reticulata* as a supplier of supplemental nutrients for crop species such as tomatoes as a possible solution to the buildup of this unusable seaweed species, which is usually left to decompose over time; this decomposition subsequently causes unpleasant odors and a decline in the aesthetics of and recreational activities on beaches in tourist areas [4, 5]. However, seaweed species are being utilized to meet future demands for fuel, food, and raw materials [6–11].

In many industries, seaweed species are utilized for their valuable contents and have been identified as natural sources of macro- and micronutrients, plant growth-promoting hormones, and vitamins. In marine habitats, seaweed species are high in mineral contents and can absorb various minerals. The macro- and micronutrients vary in seaweed species [12], and the contents of these nutrients can fluctuate. Nutrient contents are affected by several environmental factors, such as water temperature, salinity, light, and the contents of nutrients present in the environment [13]. Most environmental parameters vary according to changes in ecological conditions that can stimulate or inhibit the biosynthesis of different nutrients [14]. The abundance of essential nutrients in seaweed species makes them potential organic fertilizers. The application of seaweed is a subject of interest in agriculture, which promotes sustainable agriculture. Promoting seed germination and improving yield and production are examples of the beneficial effects of applying seaweed extracts to crop species [15, 16].

*U*. *reticulata*, a green seaweed (Chlorophyta), is considered harmful when abundant or when there is a mass proliferation of this species in coastal areas. In seagrass areas, the presence of seaweed species has disrupted ecosystem balance, as both types of organisms use the same shared resources, such as nutrients and space [17]. Rather than simply being unusable disturbance to the seagrass ecosystems, *U*. *reticulata* could be beneficial as a material for the production of compost and fertilizer. When removed from these ecosystems, the ecological balance can be maintained. As a first step, we evaluated the utilization of seaweed to enhance the growth and yield of a tested crop species-tomato (*Solanum lycopersicum*). Tomato fruits are in demand worldwide, but their supply is insufficient (according to FAOSTAT [18]). In Malaysia, tomato fruits are considered among seven vegetables for which the supply is adequate-enough to meet 126.5% of domestic needs [19]. Although adequate in supply, the amount of tomato fruits produced locally is not sufficient to meet domestic demands: 60% of tomato fruits used in Malaysia are imported from Thailand [20]. The price of tomato fruits has been increasing, from RM3.50 per kilogram to RM6.00 per kilogram, nearly double [21]. This study examines a possible method for local farmers to grow tomato plants and produce fruits via organic supplements from an unusable seaweed species, probably resulting in an extension of crop production and reducing the dependence on imports. Moreover, this method is an alternative solution to reduce chemical fertilizer dependency to promote sustainable crop production and provide ecological balance.

## Materials and methods

### Collecting and preprocessing of *U*. *reticulata* samples

*U*. *reticulata* samples were collected from the Merambong seagrass meadow (1°20’8.29" N, 103°36’17.10" E, Fig 1) in the Sungai Pulai estuary, Gelang Patah, southwestern Johor, Malaysia, in January, March, April, and May of 2017. Near the Forest City, *U*. *reticulata* was proliferating extensively and accumulated in the Merambong seagrass meadow (Fig 1). The seaweed was identified by its irregularly shaped thalli, its light-to-dark green color, and its ability to form thin, sheet-like masses of perforated blades that were a few centimeters to approximately a meter in length. *U*. *reticulata* samples were handpicked, washed using seawater to remove attached particles, and brought to the laboratory for further processing according to the methods of Selvam and Sivakumar [21]. The samples were then washed with tap water, followed by distilled water, allowed to dry in the shade for four days, and oven-dried at 60°C until a constant weight was achieved. The dried *U*. *reticulata* material was ground to a powder and kept in airtight plastic bottles at room temperature until further analysis.

### Analysis of *U*. *reticulata* mineral contents

*U*. *reticulata* samples obtained for four consecutive months from January to May were analyzed to measure their macro- and micronutrient contents. The macronutrients comprised total nitrogen (N), phosphorus (P), potassium (K), calcium (Ca), and magnesium (Mg), and the micronutrients consisted of copper (Cu), zinc (Zn), manganese (Mn), iron (Fe), and boron (B).

The total N in *U*. *reticulata* samples was determined by the combustion method (1370°C) using a CNS analyzer (LECO Corporation CNS-2000), in which dried fine and solid samples were converted to a gaseous state and measured by an infrared detector [22, 23]. The total N was expressed as a percentage of total biomass.

Total P, K, Ca, Mg, Cu, Zn, Mn, Fe, and B from *U*. *reticulata* were extracted using the dry ash method according to Korn et al. [24]. A 0.5 g plant sample was weighed in a crucible, placed in a muffle furnace, and heated at 550°C for 4 h until white or grayish-white ash was obtained; the crucible was removed from the furnace after 12 h of cooling. The ash in the crucible was moistened with a few drops of deionized water, after 5 mL of 10% HCL acid was added followed by 10 mL of 20% HNO_3_. The crucible was covered with a lid and placed in a water bath for 30 minutes. Afterward, the solution was transferred to a 100 mL volumetric flask via a filter funnel. The crucible was rinsed several times to ensure that all the solution was transferred into the volumetric flask, after which the solution was brought to 100 mL with deionized water. The flask was subsequently capped with parafilm and shaken, and the solution was then filtered through No. 2 Whatman filter paper. The digested samples were analyzed by atomic absorbance spectrophotometry (AAS Perkin Elmer 5100 PC) for nine macro- and micronutrients, excluding total B, which was quantified using inductively coupled plasma atomic emission spectroscopy (ICP-AES; Optima 8300). The total nutrient contents were expressed in grams per kilogram, milligrams per kilogram, or grams per 100 grams. All the analyses were conducted in triplicate.

### Preparation of *U*. *reticulata* extracts

Owing to their high nutrient contents as revealed from profiling, the *U*. *reticulata* samples collected in May were used for seaweed extract preparation. Two *U*. *reticulata* crude extracts were prepared: Extract “A” was prepared from dried *U*. *reticulata*, and Extract “B” was prepared from fresh *U*. *reticulata*. Crude Extract “A” of dried *U*. *reticulata* was prepared following the methods described by Rama Rao [25]. Dried *U*. *reticulata* was added to distilled water at a ratio of 1:20 (w/v, e.g., 100 g in 2 liters of distilled water), after which the solution was mixed and then autoclaved at 121°C at 15 psi for 30 minutes. The autoclaved extract was filtered through a double-layered muslin cloth and allowed to cool to room temperature (24 ± 2°C). The filtrate was collected and considered 100% *U*. *reticulata* Extract “A”. The filtrate was further diluted with distilled water to concentrations of 5%, 10%, 20%, 30%, 40%, and 50%. Crude Extract “B” of *U*. *reticulata* was prepared according to the methods of Bhosle et al. [26]. One kilogram of fresh *U*. *reticulata* was ground using an electric blender (Panasonic MX-GM1011), added to 1 L of distilled water, and then boiled for 1 h in a water bath (100°C). The resulting extract was then filtered through a double-layered muslin cloth and allowed to cool to room temperature (24 ± 2°C). The filtrate was collected as stock liquid Extract “B”, whose concentration was 100%. Then, the stock liquid was diluted with distilled water to concentrations of 5%, 10%, 20%, 30%, 40%, and 50%. Both liquid Extracts “A” and “B” were stored at 4°C until further use [27].

### Greenhouse tomato plant experiments

**Fig 2:**
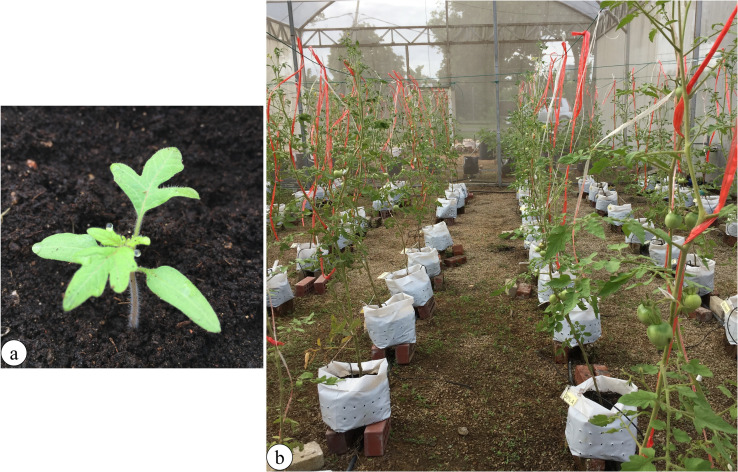
(a) A fifteen-day-old seedling grown in a tray and placed within a polybag. (b) Arrangement of experimental units in the plant growth facility at University Agriculture Park, UPM.

Tomato (*S*. *lycopersicum* L. cv. Moneymaker) seeds pretreated with 5% *U*. *reticulata* Extract “A” (the percentage of which was determined based on seed germination assessments conducted before this experiment) were sown on peat moss media in seedling trays for germination until they were 15 days old (Fig 2A). Sixty-five robust seedlings were selected: they were randomly assigned to treatment groups (Fig 2B), with five plants per treatment, and transplanted into individual 34 cm diameter x 45 cm tall polybags containing 4 kg of compost, as indicated in Table 1. One hundred milliliters of liquid *U*. *reticulata* Extract “A” and Extract “B” were applied at concentrations of 5%, 10%, 20%, 30%, 40%, or 50% per plant weekly for all 12 treatments. The control plants were grown in the same media but supplied distilled water without the *U*. *reticulata* extracts (Table 2). Daily irrigation events occurred at 800 h, 1100 h, and 1700 h with 250 mL of water for every irrigation cycle. The experiments were conducted in the plant growth facilities of University Agriculture Park, Universiti Putra Malaysia (UPM), Selangor, Malaysia. The temperature ranged from 25°C to 32°C, with 50% to 70% relative humidity during the entire growth stage of the tomato plants. The pH of the planting media ranged from 6 to 7. Tomato plants were grown for 12 weeks, and plant height at the vegetative and reproductive stages was measured weekly from the soil surface in the polybag to the tip of the plant using a measuring tape [28]. The development of tomato plants (flowering, early fruiting, and harvesting) and yield was recorded weekly.

**Table 1:** Composition of the medium and nutrient contents in which tomato plants were grown (source: University Agriculture Park, UPM).

Sources	Composition (plants and animal origins)		
	Dried/wet materials	:	Dried leaves and grass blades
		:	Leftover vegetables and fruits and
	Animal-based organic materials	:	cow, goat, and chicken dung
	Others	:	Leftover mushroom block and molasses
Nutrient contents			
	Total C (%)	:	32.09
	Total N (%)	:	0.73
	C:N ratio	:	43.96
	Total P (%)	:	0.14
	Total K (%)	:	0.43
	Total Ca (%)	:	0.98
	Total Mg (%)	:	0.26

**Table 2:** Summary of *U*. *reticulata* treatments applied to *S*. *lycopersicum* plants.

Treatment	Polybag 34 D x 45 H cm and UPM compost	Concentration (%) of the crude extract	Volume of *U*. *reticulata* extract and distilled water
Control	4 kg	0	100 mL of distilled water
*U*. *reticulata* Extract “A”	4 kg	5	5 mL of *U*. *reticulata* Extract “A” + 95 mL of distilled water
	4 kg	10	10 mL of *U*. *reticulata* Extract “A” + 90 mL of distilled water
	4 kg	20	20 mL of *U*. *reticulata* Extract “A” + 80 mL of distilled water
	4 kg	30	30 mL of *U*. *reticulata* Extract “A” + 70 mL of distilled water
	4 kg	40	40 mL of *U*. *reticulata* Extract “A” + 60 mL of distilled water
	4 kg	50	50 mL of *U*. *reticulata* Extract “A” + 50 mL of distilled water
*U*. *reticulata* Extract “B”	4 kg	5	5 mL of *U*. *reticulata* Extract “B” + 95 mL of distilled water
	4 kg	10	10 mL of *U*. *reticulata* Extract “B” + 90 mL of distilled water
	4 kg	20	20 mL of *U*. *reticulata* Extract “B” + 80 mL of distilled water
	4 kg	30	30 mL of *U*. *reticulata* Extract “B” + 70 mL of distilled water
	4 kg	40	40 mL of *U*. *reticulata* Extract “B” + 60 mL of distilled water
	4 kg	50	50 mL of *U*. *reticulata* Extract “B” + 50 mL of distilled water

### Yield and fruit quality determination

**Fig 3:**
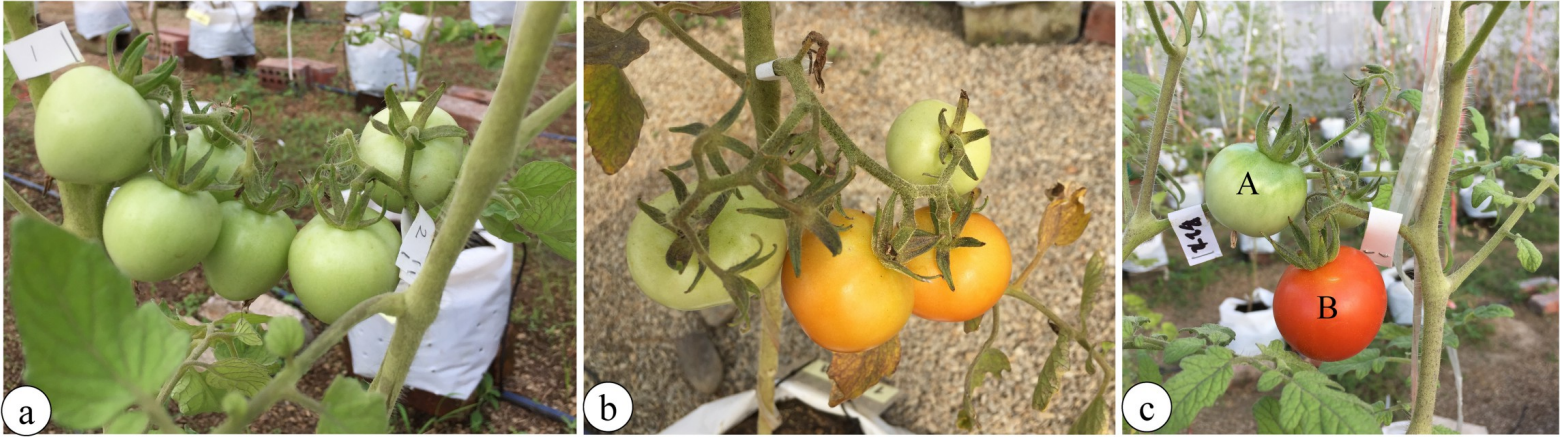
Tomato fruits. (a) Six fruits per peduncle, (b) 4 fruits, and (c) the differentiation of unripe fruit (A) and ripe fruit (B).

The yield of the tomato plants was determined based on the weight of the total number of tomato fruits per plant in each treatment (Fig 3). According to a ripeness index, yield parameters were separated for ripe and unripe tomato fruits [29]. Yield quality was evaluated for the harvested ripe tomato fruits based only on the physicochemical and analytical parameters; the physicochemical parameters included fruit firmness, total soluble solids (TSS) content, total titratable acidity (TTA), and ascorbic acid content.

The firmness of the tomato fruits was determined by measuring the force required to penetrate through the tomato flesh (skin and pericarp) by an Instron Penetrometer 545 [30]. The tomato fruit pulp was gently removed and placed over the plunger tip, where a 10 N load cell was used with a 1.6 mm flat-ended cylindrical probe at a deformation speed of 5 mm minute^-1^. The values were expressed in Newtons (N) per millimeter. The TSS content of the tomato fruits was determined using an ATAGO hand refractometer. A drop of tomato juice was placed on the prism of the refractometer, and then the reading was recorded in °Brix [31]. The TTA (expressed in grams per liter) was determined by titrating 10 g of a homogenized tomato juice after it was diluted with 50 mL of distilled water and 0.1% NaOH solution with a pH of 8.17 [32]. The ascorbic acid content of the tomato fruits was measured following the methods of Tareen et al. [33]. The procedure involved mixing 5 g of homogenized fruit pulp mixture and 5 mL of a 0.1% HCl solution (w/v) and followed by centrifugation for 10 minutes at 10,000 rpm, after which the supernatant was collected. The supernatant absorbance was measured using a spectrophotometer (SP 3000 Plus) at a wavelength of 243 nm.

Lycopene and beta-carotene contents (expressed in milligrams per 100 mL of the sample) were evaluated according to the methods used by Nagata and Yamashita [34]. One gram of tomato tissue was placed into a test tube, after which acetone and hexane were added at a 4:6 ratio, and the mixture was homogenized. The optical density of the homogenized mixture was measured at 663, 645, 505, and 453 nm wavelengths using a spectrophotometer (SP 3000 Plus). The values of lycopene and beta-carotene were calculated by the following formulas:

$$\text{Lycopene}\;\left(\text{mg}/100\;\text{mL}\right)=-0.0458{\text{A}}_{663}+0.204{\text{A}}_{645}+0.372{\text{A}}_{505}-0.0806{\text{A}}_{453}$$

$$\text{Beta-carotene}\;\left(\text{mg}/100\;\text{mL}\right)=0.216{\text{A}}_{663}-1.22{\text{A}}_{645}-0.304{\text{A}}_{505}+0.452{\text{A}}_{453}$$

### Statistical analysis

The means and standard errors of the macro- and micronutrients contents and the tomato plants’ height, yield, and fruit quality were statistically analyzed using SPSS version 20 (SPSS, Inc., USA). The macro- and micronutrient contents of *U*. *reticulata* were compared with those of other seaweed species, i.e., *Caulerpa lentillifera*, *Caulerpa racemosa*, *Kappaphycus alvarezii*, *Sargassum mcclurei*, *Hypnea valentiae*, *Porphyra crispata*, *Laurencia obtusa*, *Gracilaria tenuistipitata*, and *Gelidiella acerosa*, the results of which were subjected to principal component analysis (PCA). The ordinal relationships between the nine macro- and micronutrients (except for the element B) of *U*. *reticulata* and the other seaweed species were based on the Pearson method using XLSTAT software version 2018.1 (Addinsoft, New York, USA). Two-way ANOVA was performed to determine if the tomato plants treated with *U*. *reticulata* Extract “A” and Extract “B” showed positive growth with time compared with that of the control plants [35]. The initial ANOVA test indicated significant differences between the five Extract “A” and Extract “B” treatments and the controls (p < 0.05). A post hoc Dunnett’s test (set as treatment *>* control; p < 0.05) was used to determine if the individual treatment resulted in increased plant growth compared with that of the control [35]. The fruit production per plant of plants treated with *U*. *reticulata* Extract “A”, plants treated with Extract “B”, and the control plants were compared via one-way ANOVA and Dunnett’s test (set as production by *U*. *reticulata* treatment > control, p < 0.05). The quality parameters, fruit firmness, TSS content, TTA, ascorbic acid content, lycopene content, and beta-carotene content of ripe fruits from plants treated with Extracts “A” and “B” were compared. The relationships between the parameters were determined using PCA (XLSTAT software version 2018.1, Addinsoft, New York, USA). In addition, multiple correlation analysis was performed to determine the relationships between the quality of vine-ripened tomato (*S*. *lycopersicum*) fruits.

## Results and discussion

### Nutrient contents in *U*. *reticulata*

The contents of the macronutrients assessed in the present study were significantly different across the four months, except for the N content (Table 3). The total N ranged from 3.49% to 4.99%, which was considered high and associated with the high crude protein of *U*. *reticulata* [13]. The total P in *U*. *reticulata* ranged from 0.69 to 0.89 g kg^-1^ and was significantly highest in January 2017. Moreover, there was a significant difference in total K in the *U*. *reticulata* samples across the months, where the highest total K (7.26 g kg^-1^) was recorded in May 2017. The total Ca in *U*. *reticulata* was between 11.05 and 13.78 g kg^-1^, and there was variation across the months, with the highest recorded in January and May 2017. The total Mg in *U*. *reticulata* was lowest (12.53 g kg^-1^) in January, significantly increased (12.98 g kg^-1^) in March 2017, and slightly decreased in the following months.

**Table 3:** Macronutrients and micronutrients of *U*. *reticulata* in this study and seaweed species in other studies.

	Macronutrients					Micronutrients					
Seaweed	Total N (%)	Total P (g kg^-1^)	Total K (g kg^-1^)	Total Ca (g 100 g^-1^)	Total Mg (g kg^-1^)	Total Cu (mg kg^-1^)	Total Zn (mg kg^-1^)	Total Mn (mg kg^-1^)	Total Fe (g kg^-1^)	Total B (mg kg^-1^)	Reference
1. *Ulva reticulata* (January)	3.49 ± 0.00^a^	0.89 ± 0.00^a^	4.18 ± 0.02^c^	13.78 ± 0.15^a^	12.53 ± 0.04^c^	6.58 ± 1.19^b^	36.30 ± 1.76^a^	1394.54 ± 18.23^a^	2.85^a^	30.15 ± 2.58^c^	
2. *U*. *reticulata* (March)	4.99 ± 0.05^a^	0.69 ± 0.00^c^	6.31 ± 0.13^b^	12.52 ± 0.34^b^	12.98 ± 0.03^a^	6.35 ± 0.59^b^	19.92 ± 0.52^c^	206.72 ± 38.86^d^	2.21^c^	43.42 ± 0.81^b^	*In this study
3. *U*. *reticulata* (April)	4.20 ± 0.03^a^	0.76 ± 0.02^b^	6.91 ± 0.30a^b^	11.05 ± 0.18^c^	12.78 ± 0.03^b^	17.55 ± 1.15^a^	28.04 ± 0.75^b^	476.31 ± 6.81^c^	2.39^b^	57.67 ± 2.20^a^	
4. *U*. *reticulata* (May)	3.99 ± 0.01^a^	0.77 ± 0.02^b^	7.26 ± 0.35^a^	13.61 ± 0.19^a^	12.79 ± 0.03^b^	15.08 ± 1.21^a^	30.53 ± 0.69^b^	777.58 ± 10.75^b^	2.91^a^	50.86 ± 0.44^a^	
5. *U*. *reticulata*	0.04	0.61	12.00	19.40	38.50	8.61	24.41	392.51	0.99	-	[37]
6. *U*. *reticulata*	3.37	1.80	15.40	1.40	1.40	0.60	33.00	481.00	1.75	-	[38]
7. *Caulerpa lentillifera*	1.99	10.30	9.70	7.80	6.30	22.00	26.00	79.00	0.09	-	[38]
8. *C*. *racemosa*	0.93	1.20	36.10	59.20	14.50	2.73	12.22	5.02	0.15	-	[37]
9. *Kappaphycus alvarezii*	0.03	0.17	19.70	2.28	2.87	1.48	3.07	12.36	0.05	-	[16]
10. *K*. *alvarezii*	0.47	0.91	22.50	6.80	13.30	9.93	21.24	5.44	0.19	-	[37]
11. *Sargassum mcclurei*	1.30	0.82	12.50	97.60	17.80	10.92	33.92	159.42	1.30	-	[37]
12. *Hypnea valentiae*	0.89	1.13	14.10	81.30	50.20	3.12	48.02	10.42	0.26	-	[37]
13. *Porphyra crispata*	2.29	2.04	10.50	20.20	8.60	3.72	40.83	17.43	0.37	-	[37]
14. *Laurencia obtusa*	0.71	0.62	64.20	46.30	26.10	4.63	8.01	13.61	0.10	-	[37]
15. *Gracilaria tenuistipitata*	0.05	1.11	10.80	10.40	5.80	14.92	37.13	1571.11	0.13	-	[37]
16. *Gelidiella acerosa*	1.01	0.61	27.30	79.10	8.20	3.64	23.33	5.43	0.01	-	[37]

*The data are the mean values ± standard errors, and different letters within columns are significantly different (Tukey’s test, p < 0.05).

The micronutrient contents in *U*. *reticulata* are presented in Table 3. The total Cu in *U*. *reticulata* ranged from 6.35–6.58 mg kg^-1^ in March and January but was higher in April and May 2017–17.55 mg kg^-1^ and 15.08 mg kg^-1^, respectively. The total Zn contents recorded in January and May 2017 were 36.30 mg kg^-1^ and 30.53 mg kg^-1^, respectively. The present study’s total Mn, Fe, and B in *U*. *reticulata* varied across the months, ranging from 206.72–1394.54 mg kg^-1^, 2.21–2.91 g kg^-1^, and 30.15–57.67 mg kg^-1^, respectively. *U*. *reticulata* presented comparatively higher contents of B compared with Cu, Zn, and Fe. B is considered an essential element for plant growth and development. The main functions of B are related to cell wall strength and development, plant reproduction, N fixation, and plant metabolism [36]. Due to a lack of information on B in other seaweed species, PCA was performed while B was excluded.

In seaweed, the chemical constituents change with the season, environmental condition, and development and growth [14]. Thus, investigating the variation in chemical contents in seaweed is necessary for future applications, such as nutrient supplementation for plants. Based on the macro- and micronutrient contents of *U*. *reticulata* investigated in this study, the samples in May 2017 were selected for further application and testing on crop plants.

**Fig 4:**
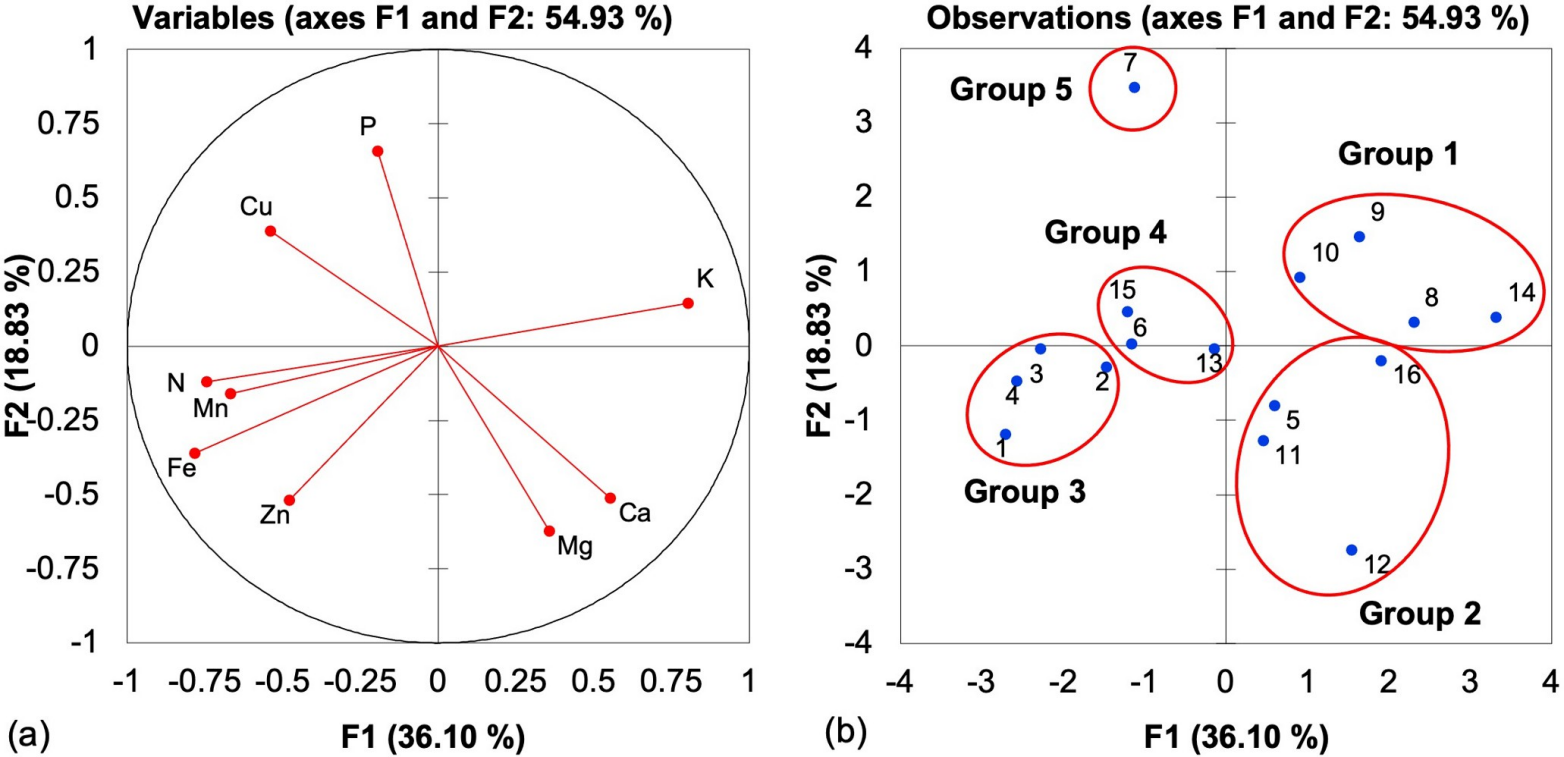
(a) Plot of the variables tested for macronutrients and micronutrients of seaweed species, where the percentage in the parentheses represents the variation of each component. (b) Positions of the PC scores of the 16 seaweed species (listed in Table 3) for macronutrients and micronutrients according to F1 and F2.

The macro- and micronutrient contents of *U*. *reticulata* were compared with those of other seaweed species, as shown via PCA in Fig 4A and 4B. The first two principal components (PCs) accounted for 54.93% of the total variance. PC1 explained a high percentage of the total variance compared to 18.83% of the total variance explained by PC2. PCA indicated that the different macro- and micronutrients of *U*. *reticulata* and other seaweed species could be clustered into five groups. Group 1 consisted of *C*. *racemosa*, *K*. *alvarezii* [16], *K*. *alvarezii* [37] and *L*. *obtusa*. These seaweed species have a high content of K; K in seaweed assists in water regulation and photosynthesis and improves meristematic growth, photosynthate translocation, and disease resistance [39]. Group 2 included *U*. *reticulata* [37], *S*. *mcclurei*, *H*. *valentiae*, and *G*. *acerosa*, which presented higher values of Mg and Ca. In comparison, the total Mg in *U*. *reticulata* in the present study was eight times higher than that in the study by Ratana-arporn Chirapart [38] and three times lower than the content reported in [37]. Mg is an important component of chlorophyll and in various enzymes and constituents [40]. Ca in seaweed aids in enzyme activation, cell elongation, and cell stability. Seaweed is a rich source of secondary nutrients such as Mg, which function in photosynthesis, phloem export, root growth, and N metabolism [39].

*U*. *reticulata* (sampled across all four months-January to May) was assigned to group 3, whose members have higher N, Mn, Fe, and Zn contents. *U*. *reticulata* in the present study contained 87 times more N than the same species reported in [37]. Similarly, a lower N content was reported by Ratana-arporn and Chirapart [38]. N is an essential macronutrient and a major component of many essential organic compounds required for normal plants. It is a constituent of cell protoplasm, amino acids, proteins, nucleic acids, and chlorophyll. Some proteins act as enzymes involved in the catalysis of biochemical reactions in plants [40, 41]. Thus, *U*. *reticulata* in this study could potentially supply N to plants. Mn is a component of several cation-activated enzymes, such as decarboxylases, kinases, and oxidases, and is essential for the formation of chlorophyll, the reduction of nitrates, and respiration [40].

Group 4 consisted of *U*. *reticulata* [38], *P*. *crispata*, and *G*. *tenuistipitata*, with no single variable correlated with macro- or micronutrients among the seaweed species. Group 5 comprised *C*. *lentillifera*, with the highest P and Cu contents. In the present study, the P content in *C*. *lentillifera* was approximately 15 times higher than that in *U*. *reticulata*. According to Pedersen et al. [42], seaweed species such as *Ulva* spp. have a thin structure, grow fast, and take up dissolved inorganic P much faster than thicker, slower-growing species such as *Fucus*, *Ascophyllum*, and *Laminaria*; however, species like *Ulva* spp. also have higher P demands per unit biomass and time. *U*. *reticulata* in this study had the lowest P, which was probably attributed to the water salinity level of 28–32. The P uptake is higher at lower salinity levels and lower at higher salinity levels [43]. When applied to plants, P from seaweed increases root development and increases the root:shoot ratio, thereby promoting sufficient absorption of nutrients from deeper soil layers and influencing crop maturity. P is also an important constituent of nicotinamide dinucleotide (NADP), the niacin component of the vitamin B complex, which helps photosystem I produce NADPH [39]. According to Ratana-arporn and Chirapart [38], the high P content in this species may be a potential source of supplemental minerals for plants. The total Cu content in the seaweed examined in this study agreed with the findings of earlier studies by Ratana-arporn and Chirapart in which the same species were used [38]. According to Turan and Ko¨se [44], seaweed extracts were more effective at promoting Cu uptake in grapevine than the nutrient element level of the growth media was due to increased membrane permeability of root, leaf, and stoma cells and the hormone-like activities of the seaweed extract exerted through its participation in plant metabolic activity.

Plants can utilize the mineral contents in seaweed as essential nutrients for normal physiological functions, such as for development, growth, yield, and defense mechanisms against pests and diseases [41]. The mineral nutrient composition of plants is therefore important for controlling plant physiological and biochemical processes. Nutrient deficiencies may lead to changes in these processes and may disrupt plant growth and yield [36]. The abundance of nutrients in *U*. *reticulata* makes them suitable for further utilization as organic supplemental plant nutrients.

### Effects of *U*. *reticulata* extracts on the growth, development, and yield of tomato plants

**Fig 5:**
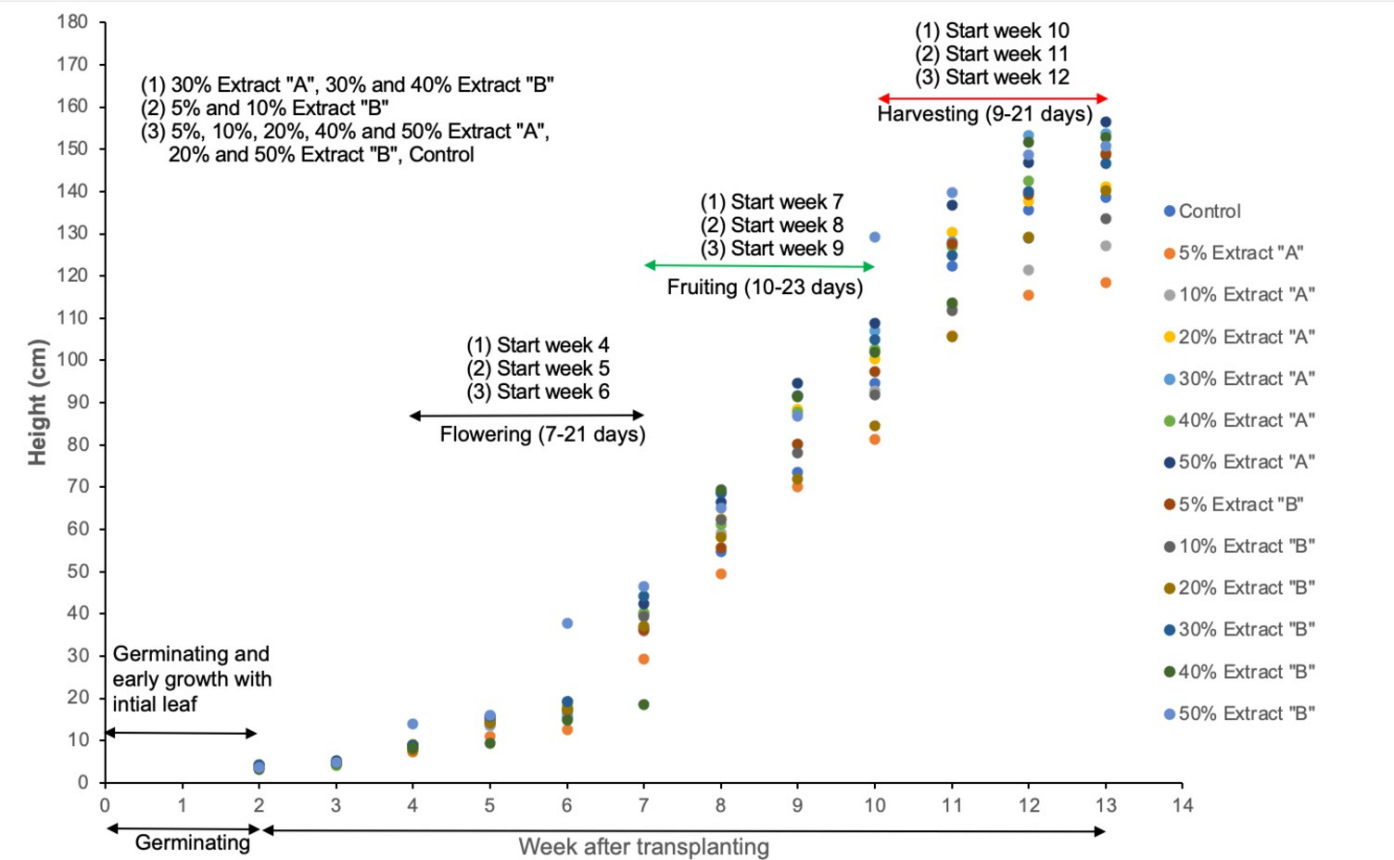
*S*. *lycopersicum* plants treated with 30% and 50% Extract “A” and Extract “B” were taller than the control plants from week 9 to week 12 (two-way ANOVA, Dunnett’s test, treatment > control, p < 0.05).

The results showed that, compared with the control treatment, 30% Extracts “A” and “B” of the seaweed *U*. *reticulata* and 50% Extracts “A” and “B” (two-way ANOVA, Dunnett’s test, set as treatments > control, p < 0.05) promoted more tomato growth. In addition, plant height significantly increased from week 9 to week 12 (Fig 5). Similar studies have shown positive effects on plant height in response to the application of seaweed extracts, e.g., the tallest potato plants occurred in response to the application of 10% *Gracilaria edulis* sap as a recommended dose of fertilizer [45], and soybean (*Glycine max*) plant height increased with increasing concentrations of *K*. *alvarezii* (Doty) extract [16]. Sunarpi et al. [46] tested the effects of extracts of many seaweed species, and those only from *Sargassum* spp., *U*. *reticulata*, and *Hydroclathrus* spp. increased the height of rice plants. The application of seaweed extracts improves the absorption of nutrients through the roots, promoting additional biomass and overall strong plant growth.

The plants treated with all Extract “A” and Extract “B” began flowering at Week 4 (30% Extract “A”, 30% and 40% Extracts “B”). The flowering of the plants under the other treatments occurred later—from Week 5 (5% and 10% Extract “B”) until Week 6 (5%, 10%, 20%, 40%, 50% Extract “A” 20%, and 50% Extract “B”, as well as the controls, within 7–21 days). Fruiting started during Week 7 through Week 9 for the same treatments as those above, within 10–23 days, and mature fruits were ready for harvest beginning Week 10 until Week 12, within 9–21 days (Fig 5).

Jones [48] mentioned that the duration of each stage depends on the variety and other environmental factors, such as air temperature, light conditions, soil conditions, and nutrients. The five growth stages of tomato described by Jones [47] are as follows: germination and early growth of initial leaves (between 25 and 35 days), vegetative growth (20 to 25 days), flowering (20 to 30 days), early fruiting (20 to 30 days), and mature fruit production (15 to 20 days). The data in this study agree with the average duration to reach the mature fruiting stage (from transplanting) for most greenhouse tomato varieties: depending on variety differences with respect to maturity level, ripeness occurs between 65 and 100 days, with a minimum of 75 days from transplanting reportedly needed to reach the first harvest for most cultivated tomato fruits [47]. Tomato plants treated with 30% Extract “A”, 30% and 40% Extract “B” exhibited enhanced development associated with the presence of macro- and micronutrients [16]. The readily available nutrients in the extracts could promote the efficient absorption of nutrients and their subsequent transport, enhancing plant development and hastening the time needed for flowering, fruiting, and harvesting. *U*. *reticulata* extracts have dual functions, i.e., they improve plant vegetative growth (30% Extracts “A” and “B” and 50% Extracts “A” and “B”) and promote early flowering and fruiting (30% Extracts “A” and “B” and 40% Extract “B”). In addition, the extracts hastened the time to harvest compared to that of the control plants.

### Tomato fruit yield

Compared with the control treatment, all treatments of Extract “A” and Extract “B” (except for 50% Extract “B”) resulted in a higher total yield (one-way ANOVA, Dunnett’s test, p < 0.05; treatments > control). In addition, compared with the other treatments, 40% Extract “A” yielded 76% (80.96 g plant^-1^) more vine-ripened fruit and 25% (26.00 g plant^-1^) less unripe fruits. Moreover, the 40% Extract “A” resulted in yields three times higher than those of the control plants. The lowest total yield (only 23.38 g plant^-1^) was recorded in response to 50% Extract “B”; this yield was lower than that of the controls (Table 4). Extract “A” generally resulted in greater total yields and total ripe tomato fruit yields than Extract “B” did, and increasing the concentration of the Extract “B” by more than 20% negatively affected yields. No specific trend was observed for the increase or decrease in Extract “A” concentration on tomato yield (vine-ripened or unripe fruit yield). The yield increases of the seaweed-treated plants were related to the rich contents of *U*. *reticulata* macro- and micronutrients essential for plant growth. The plants absorbed the nutrients supplied by the seaweed, causing the cells and tissues to grow and develop to form new organs; essentially, the plants form more shoots and leaves. In essence, plants use this sufficient supply of nutrients to form organs and tissues, resulting in increased yields.

**Table 4:** Treatment and yields of *S*. *lycopersicum* fruits.

Treatment	Vine-ripened fruits (mean yield, g plant^-1^)	Unripe fruits (mean yield, g plant^-1^)	Cumulative mean yield (g plant^-1^)
Control	32.05±0.75	3.23±0.14	35.28±0.64
*U*. *reticulata* Extract “A”			
5%	71.45±1.45	22.89±1.25	94.34±2.14
10%	49.81±1.87	39.22±2.11	89.03±2.83
20%	56.30±1.96	19.06±1.15	75.36±2.72
30%	47.54±2.23	0.24±0.02	47.78±2.21
40%	83.20±2.34	27.07±1.72	110.26±2.81
50%	82.12±1.91	9.12±0.15	91.24±1.79
*U*. *reticulata* Extract “B”			
5%	65.65±3.66	4.53±0.20	70.18±3.70
10%	53.78±1.88	8.26±0.26	62.04±2.02
20%	60.78±1.50	14.23±1.04	75.01±2.11
30%	54.69±1.69	11.03±1.20	65.72±2.65
40%	44.84±1.89	4.30±0.12	49.14±2.15
50%	15.38±0.92	4.30±0.14	23.90±0.82

Based on the yield data from this study, similar results were also observed in other crop species, where seaweed treatment resulted in increased yields of wheat (*Triticum aestivum*) upon application of 20% extracts of *Sargassum wightii* [27] and where maximum soybean (*G*. *max*) yields occurred in response to the application of 15% extracts of the seaweed *K*. *alvarezii* [16]; moreover, positive results concerning the growth and yield of rice in response to the application of aqueous extracts of *Hydroclathrus* spp. were detected [46]. However, Finnie and Van Staden [48] reported that kelp extracts at relatively high concentrations (1:100 seaweed extract:water) inhibited root growth but had a positive effect at lower concentrations (1:600), which was similar to the results observed for the fresh *U*. *reticulata* extracts in the present study.

### Tomato fruit quality

**Fig 6:**
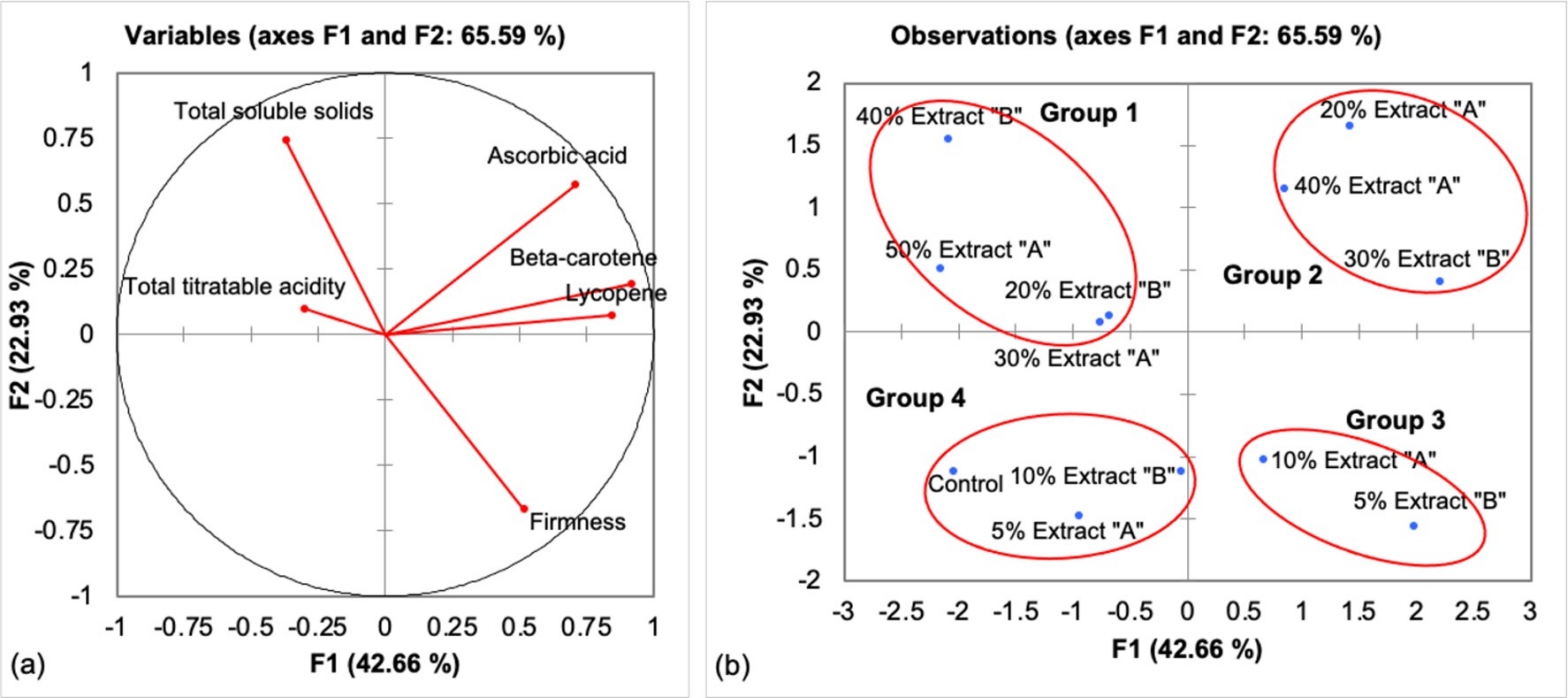
(a) Vine-ripened fruit firmness; TTA; and TSS, ascorbic acid, beta-carotene, and lycopene contents. The percentage in parentheses represents the variation of each component. (b) Positions of the PC scores of treatments according to PC1 and PC2.

Table 5 shows the treatments and quality variables of the vine-ripened tomato fruits. The PCA results concerning the TTA and the TSS, ascorbic acid, lycopene, and beta-carotene contents are shown in Fig 6. The first two PCs accounted for 65.59% of the total variance; PC1 explained a higher percentage of the total variance (42.66%) than PC2 (22.93%). Both Extract “A” and Extract “B” of *U*. *reticulata* contributed to the quality of the ripe fruits. TTA and TSS, ascorbic acid, beta-carotene, and lycopene contents were tightly associated with the positive side of PC1, whereas fruit firmness was associated with the negative side of PC1.

**Table 5:** Treatment and quality of *S*. *lycopersicum* vine-ripened fruits.

Treatment	Firmness (N mm^-1^) n = 3	Total soluble solids (°Brix)	Total titratable acidity (g L^-1^)	Ascorbic acid (mg 100 g^-1^)	Lycopene (mg 100 mL^-1^)	Beta-carotene (mg 100 mL^-1^)
Control	0.95±0.22	3.56±0.22	0.43±0.03	18.41±1.22	0.17±0.02	0.04±0.01
*U*. *reticulata* Extract “A”						
5%	1.04±0.02	3.56±0.16	0.32±0.01	20.37±0.18	0.17±0.02	0.04±0.01
10%	1.29±0.12	3.50±0.57	0.38±0.05	28.28±1.77	0.20±0.02	0.06±0.01
20%	1.02±0.13	3.96±0.75	0.38±0.08	31.26±0.62	0.37±0.12	0.08±0.02
30%	1.12±0.33	4.08±0.15	0.35±0.01	22.87±0.24	0.27±0.05	0.04±0.00
40%	0.89±0.06	3.84±0.20	0.35±0.04	27.88±2.13	0.45±0.05	0.07±0.00
50%	1.01±0.10	4.20±0.42	0.37±0.04	21.38±0.78	0.12±0.01	0.04±0.01
*U*. *reticulata* Extract “B”						
5%	1.44±0.30	3.63±0.82	0.36±0.04	23.62±0.75	0.52±0.05	0.07±0.00
10%	1.24±0.11	3.63±0.18	0.39±0.03	23.62±0.14	0.25±0.06	0.06±0.01
20%	1.13±0.17	4.00±0.13	0.38±0.03	23.75±0.73	0.28±0.04	0.05±0.00
30%	1.07±0.13	3.70±0.17	0.37±0.01	27.41±2.45	0.63±0.10	0.08±0.01
40%	0.74±0.05	4.06±0.16	0.40±0.02	24.83±0.14	0.10±0.01	0.04±0.00
50%	-	-	-	28.62±0.00	0.31±0.06	0.10±0.01

Treatments and fruit quality were assigned to four groups. Group 1, with 30% and 50% Extract “A”, 20% and 40% Extract “B” produced fruits with a high TTA and a high TSS content. The sour taste of tomato fruits is strongly correlated with TTA and decreases in acidity levels have been associated with quality loss during postharvest tomato storage. Variation in TTA is greater in large tomato fruits than in small tomato fruits [49]. TTA and TSS contents can influence consumer preferences. The TSS content, measured as °Brix, is an indicator of sweetness, although sugars are not the sole soluble component this parameter measures. According to Murtic et al. [50], yield and quality characteristics such as TSS, vitamin C, and lycopene contents of cherry tomato seedlings treated with seaweed extract increased under both control and drought-stress conditions compared to those of untreated plants under identical conditions. Sutharsan et al. [51] further reported that the TSS content of tomato fruit increased compared with that of the controls in all treatments except relatively high concentrations (100%) of seaweed liquid extracts (SLEs) of *Sargassum crassifolium*. The TSS content depends mainly on the transport of ions and organic solutes, and most are converted to glucose within fruits [51]. It is known that an increase in the content of TSS (sugars, amino acids, and organic acids) is influenced by water deficit, in which major compounds accumulate in the fruits. A high content of TSS increases the quality of fresh fruit, positively affecting their flavor, taste, and water content [52]. The increase in TSS contents during fruit ripening is due to the conversion of starch to sugars, and various cultivars present different TSS contents due to inherent differences in their genetic characteristics.

Group 2 consisted of treatments including the 20% and 40% Extract “A” and 30% Extract “B”, which resulted in the production of fruits with relatively high ascorbic acid, beta-carotene, and lycopene contents. Ascorbic acid, known as vitamin C, benefits plants as it promotes biotic and abiotic stress tolerance, and improves postharvest fruit quality. The ascorbic acid content in plant cells is influenced by the regulation of its synthesis, metabolic recycling and degradation, and transport [53]. The major factor contributing to variation in the ascorbic acid content is the growth environment. Specifically, light was determined to be a key factor affecting ascorbic acid content [54]. In the present study, we recorded ascorbic acid contents in the treated plants (27.41–31.26 100 g^-1^) that were 1.5 times higher than those of the control plants and higher than those obtained by Matthews et al. [54], whose ascorbic acid content ranged from 10.7–20.9 mg 100 g^-1^. Other studies indicated that plants treated with a relatively high concentration of brown seaweed extract (10% *Sargassum johnstonii* extract) produced tomato fruits with the maximal amount of ascorbic acid [55]. According to the human recommended daily allowance (RDA), the recommended ascorbic acid content set by the Food and Drug Administration (FDA) is 60 mg [54]. Thus, approximately 200 g of tomato treated with 20% Extract “A” in the present study can supply the amount of ascorbic acid needed by the human body. The high amount of ascorbic acid in the fruits in the present study suggest that either fresh tomato fruits or processed tomato fruits can be consumed to meet dietary needs, excluding the possibility that the amount of ascorbic acid may be slightly reduced during canning.

Carotenoids include both lycopene and beta-carotene. Lycopene is a bright red carotene with an antioxidant ability that is twice as high as that of beta-carotene, which aids in the quenching of free radicals. Lycopene is localized in chromoplasts of tomato fruits; the pigmentation of red ripe tomato fruits is determined by the synthesis of carotenoids, lycopene, and beta-carotene [56]. The degree of redness is directly proportional to the concentration of lycopene; extracts of *Ascophyllum nodosum* seaweed improved the yield and quality of cherry tomato fruits, including the lycopene content under both standards and drought-stress conditions compared to control conditions [50]. Seaweed extracts may play a role in the translocation of cytokinin from the roots to developing fruits or may even increase cytokinin synthesis directly within the fruits [55]. Given that there are cytokinin-like substances present in the aqueous extracts of seaweed (*U*. *reticulata*), mobilization of nutrients to the fruits may increase the lycopene concentration. Seaweed extracts can rapidly supply nutrients to plants, as several studies mentioned the possibility of active uptake through plants’ roots and root hairs.

Beta-carotene is a provitamin [56] and one of the highly unsaturated hydrocarbon carotenoids widely distributed in fruits and vegetables. In the present study, under the seaweed treatments, there was a significantly higher beta-carotene content in the tomato fruits than that of the control fruits—0.07–0.08 mg 100 mL^-1^ compared to 0.04 mg 100 mL^-1^, respectively, which is nearly double. A similar observation was reported by Sidhu et al. [56], where the application of *A*. *nodosum* seaweed extract on tomato plants resulted in increased beta-carotene contents in the fruits.

Group 3 represented a cluster of low-concentration treatments (10% Extract “A” and 5% Extract “B”), which resulted in the production of higher fruit firmness. Based on the work of Trejo et al. [15], *Bryothamnion triquetrum* seaweed extracts did not affect the firmness of fruits from tested cucumber (*Cucumis sativus* L.) plants.

Group 4 represented treatments with a low concentration of extracts (5% Extract “A” and 10% Extract “B”). The results were similar to those of the controls, and there were no differences in specific aspects of fruit quality.

This present study showed that treatments of particular concentrations of Extracts “A” and “B” positively affected fruit quality by significantly increasing the TTA and TSS, ascorbic acid lycopene, and beta-carotene contents in the tomato fruits.

### Correlations between vine-ripened tomato fruit quality characteristics

The correlation matrix in Table 6 shows significant and strong correlations (p < 0.05) for some of the quality characteristics of the vine-ripened tomato fruits. Beta-carotene showed a strong positive correlation with ascorbic acid (r = 0.761) and lycopene (r = 0.719) contents. Therefore, the higher lycopene, beta carotene, and ascorbic acid contents in tomato fruits are, the more intense the pigmentation of vine-ripened tomato fruits.

**Table 6:** Correlation matrix for all quality characteristics of vine-ripened tomato (*S*. *lycopersicum*) fruits.

Quality characteristic	Firmness	Total soluble solids	Total Titratable acidity	Ascorbic acid	Lycopene	Beta-carotene
Firmness	1.000	-0.449	-0.207	0.029	0.324	0.310
Total soluble solids		1.000	-0.029	0.082	-0.189	-0.270
Total titratable acidity			1.000	-0.074	-0.269	-0.098
Ascorbic acid				1.000	0.474	**0.761**
Lycopene					1.000	**0.719**
Beta-carotene						1.000

The values in bold are significant at p < 0.05.

## Conclusions

The seaweed species *U*. *reticulata* collected from Merambong seagrass meadow in the Sungai Pulai estuary of Johor contains reasonable amounts of macro- and micronutrients. Compared to extracts of other seaweed species used for plant growth promoters, *U*. *reticulata* extracts have higher N, Mn, Zn, and Fe contents. *U*. *reticulata* can potentially provide supplemental nutrients for crop production, e.g., tomato fruit production. The application of 30% Extracts “A” and “B” and 50% Extracts “A” and “B” (extracts from dried and fresh *U*. *reticulata*, respectively), significantly affected tomato plant height. However, the extract concentration that increased plant height and hastened flowering and fruiting did not increase total fruit yields. Nonetheless, both treatments that increased tomato plant height and hastened both flowering and fruiting increased TTA and TSS, beta-carotene, lycopene, and ascorbic acid contents in vine-ripened fruits. Agronomically, 40% Extract “A” application resulted in the highest total fruit yield. However, other extract concentrations, e.g., 5% Extracts “A” and “B”, 10%-20% Extracts “A” and “B”, and 50% Extract “A”, resulted in total yields that were double those of the controls. Extract “A” was better at providing supplemental nutrients with respect to total fruit yield and quality considerations.

## Supporting information

S1 Fig 4 Data for macro- and micronutrients of *U*. *reticulata*. (XLSX)

S2 Fig 5 Data *Solanum lycopersicum* plants treated with *U*. *reticulata* Extract “A” and Extract “B” compared to control. (XLSX)

S3 Fig 6 Data of firmness, total titratable acidity, total soluble solids, ascorbic acid, beta-carotene, and lycopene of tomato vine-ripened fruit quality. (XLSX)
